# Virchow’s Triad and the Role of Thrombosis in COVID-Related Stroke

**DOI:** 10.3389/fphys.2021.769254

**Published:** 2021-11-10

**Authors:** Francisco J. Gonzalez-Gonzalez, Mary Rodriguez Ziccardi, Mark D. McCauley

**Affiliations:** ^1^Division of Cardiology, Department of Medicine, College of Medicine, University of Illinois at Chicago, Chicago, IL, United States; ^2^Jesse Brown VA Medical Center, Chicago, IL, United States; ^3^Department of Physiology and Biophysics and the Center for Cardiovascular Research, College of Medicine, University of Illinois at Chicago, Chicago, IL, United States

**Keywords:** COVID-19, stroke, Virchow’s triad, thrombosis, cardiovascular

## Abstract

In December 2019, severe acute respiratory syndrome coronavirus 2 (SARS-CoV-2) was identified as a virally transmitted disease. Three months later, SARS-CoV-2 became one of the largest pandemics in recent times, causing more than 235 million cases globally, and accounting for at least 4.8 million deaths to date. SARS-COV-2 infection was initially classified as a respiratory tract infection, but later was recognized as a multisystemic disease compromising gastrointestinal, hematological, cardiac, and neurological systems. With this Review, we aim to describe the epidemiology, risk factors, mechanisms, and management of cerebrovascular events in patients infected with COVID-19. Neurological manifestations related to thromboembolic cerebrovascular events in patients infected with COVID-19 have been frequent and associated with poor prognosis in the majority of cases. A better understanding of the mechanisms of thrombosis and etiologies of this new disease process are necessary to determine how to prevent and treat patients to reduce their length of stay, morbidity, and mortality.

## Introduction

**FIGURE 1:**
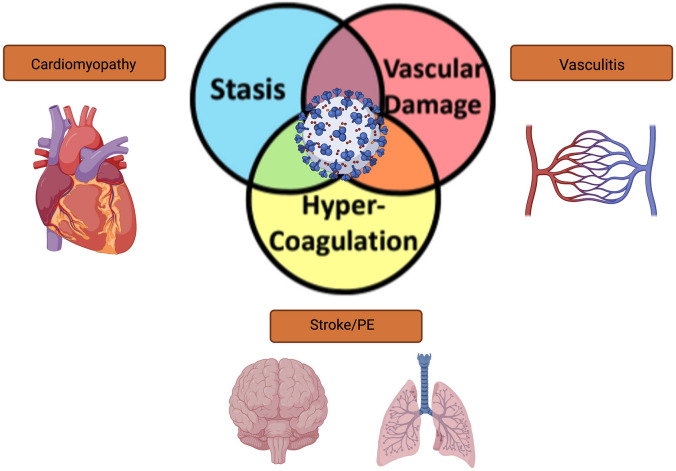
Schematic representation of the overlapping limbs of Virchow’s triad and how COVID-19 affects vascular thrombosis in different organ systems.

Severe acute respiratory syndrome coronavirus 2 (SARS-CoV-2) causes the infection commonly known as COVID-19. SARS-CoV-2 virus has infected more than 235 million people globally, causing 4.8 million deaths. The United States has had a substantial proportion of these cases, and over 44 million cases have been reported to date (World Health Organization, 2021). The primary symptoms of infection for COVID-19 are fever, dry cough, dyspnea, and hypoxia, with the development of an interstitial pneumonia in more severe cases. In addition to substantial respiratory compromise and typical viral prodrome, other organ systems have been shown to be affected with COVID-19 infection, including involvement of cardiovascular, hematological, renal, gastrointestinal, and neurological systems, causing symptoms of shortness of breath, fatigue, nausea or vomiting, and diarrhea. In the most severe cases, altered mental status, chest pain, discoloration in the lips or nail beds may also occur. One unique hematologic property affecting all of the organ systems mentioned is the ability of the virus to cause intravascular thrombosis; this property occurs in contradistinction from many other viral illnesses, which promote a hemophilic or hemorrhagic state (Ahmed et al., 2020). In patients who survive acute COVID-19 infection, there is a 30% risk for the development of Post-Acute Sequelae of COVID-19 syndrome (PASC, also called long-COVID), which has been described to have lingering symptoms of fatigue (58%), headache (44%), attention deficit disorder (27%), hair loss (25%), and dyspnea (24%) (Lopez-Leon et al., 2021). Furthermore, patients previously infected with SARS-CoV-2 are 7.6 times more likely to have an ischemic stroke than people who developed other significant viral infections, such as influenza (Merkler et al., 2020). Given that both acute and chronic (PASC) infections of COVID-19 contribute to neurological dysfunction and stroke, it is imperative that the mechanisms of stroke risk in COVID-19 be determined. In this Review, we examine the current data regarding epidemiology, risk factors, mechanisms, and management of cerebrovascular events in patients infected with COVID-19. We specifically discuss risk factors for Virchow’s triad of intravascular thrombosis (Figure 1), and how COVID-19 infection satisfies all three limbs of this triad in infection-related stroke.

## Epidemiology

The incidence of stroke since the beginning of the COVID-19 pandemic was initially believed to be reduced with a lower number of admissions for acute cerebrovascular events (Markus and Brainin, 2020). However, despite this initial observation, others have more recently reported a significant increase in cumulative incidence of stroke in patients with COVID-19 infection, including both intracerebral hemorrhage and ischemic stroke (Beyrouti et al., 2020). These clinical observations led to subsequent analyses to determine the relationships between COVID-19 and stroke. The first report from the Multinational COVID-19 Stroke Study Group and a recent meta-analysis on the incidence of stroke in infected patients reported a stroke incidence rate of 0.5–1.4%, showing that the odds of stroke after COVID-19 infection might not be greater than in non-infected patients (Bekelis et al., 2020; Shahjouei et al., 2020; Yamakawa et al., 2020). A meta-analysis by Nannoni et al. (2021) reports the incidence of acute cerebrovascular events in patients with COVID-19 is 0.4–8.1%. Across the 108,571 COVID-19 patients and twenty-four observational cohort studies that were evaluated in this study, from studies performed between December 2019 and September 2020, ischemic or hemorrhagic stroke was reported in 1,106 patients yielding on meta-analysis an overall pooled incidence of acute cerebrovascular disease of 1.4% (95%CI: 1.0–1.9). The incidence was higher in Asia (3.1%; 95%CI: 1.9–5.1) than in Europe (1.2%, 0.7–1.9) and North America (1.1%; 95%CI: 0.8–1.4). Whether this difference in stroke incidence was related to genetic, environmental, or other causes is unknown and currently under investigation.

Comorbidities may play a significant role in the development of stroke in COVID-19 infection; for example, strokes in patients with COVID-19 may be due to usual known causes such as atherosclerosis, hypertension, and atrial fibrillation (Wang Z. et al., 2020). In reported cases of stroke among COVID-19 infected patients, acute ischemic stroke was more frequent than the hemorrhagic stroke (87.4% vs. 11.6%) (Nannoni et al., 2021). Ischemic events showed large vessel occlusion in 79.6% of cases, and involvement of multiple vascular territories was found in 42.5% of cases (Markus and Brainin, 2020). Dhamoon et al. (2021), in a multicenter retrospective observational study, found that 38% of patients admitted with stroke were COVID-19 positive, and these patients had a more severe stroke (mean National Institute of Health Stroke Scale of 15.5 vs. 9.6 among those without COVID-19). Additionally, strokes were more likely to be cryptogenic when compared with COVID-19 negative patients (51.8 vs. 22.3%, *p* < 0.001). These results suggest that the possible origin of stroke is not associated with large artery occlusion, cardioembolic, or small vessel occlusion, which are traditional cardiovascular stroke risk factors. Additionally, only 28.9% of ischemic strokes were proven cardioembolic in COVID-19 infected patients, that was more common the cause in ischemic stroke in COVID-19 negative patients with 42.2% (Dhamoon et al., 2021). Also, they described the anatomical location of the ischemic stroke was more likely to be lobar and related with large vessel territories (non-lobar areas: basal ganglia, internal or external capsule, thalamus, cerebellum, or brainstem).

Similar results were found in one of the largest studies from New York City during the peak of the pandemic (Yaghi et al., 2020), which retrospectively evaluated 3,556 hospitalized patients with COVID-19 infection; only 32 patients (0.9%) had neurovascular imaging proving ischemic stroke. Stroke was classified as cryptogenic in 65.6% of cases and interestingly, this rate is higher than contemporary controls and historical controls (30.4 and 25.0%, respectively, with a *p* < 0.001), and similar to was previously described by Yaghi et al. (2020), Dhamoon et al. (2021) also showed that COVID-19 patients have a higher National Institute of Health Stroke Scale (NIHSS) severity score than contemporary controls (23 vs. 12 *p* = 0.001) which indicates higher severity and poorer prognosis for COVID-19 infected patients.

In the cohort evaluated by Dhamoon et al. (2021) they found that in patients with COVID-19 infection, at the time closest to stroke, mean coagulation laboratory values showed a mild coagulopathy and elevated inflammatory markers (prothrombin time 15.4 ± 3.7 s and INR 1.4 ± 1.3, elevated white blood cells, platelets, C-Reactive protein, erythrocyte sedimentation rate and interlukin-6 levels) and 97% of these patient had an elevated d-dimer. These laboratories are suggestive that the etiology of stroke in this population is likely secondary to endothelial inflammation and coagulopathy. Additionally, they observed that patients infected with COVID-19 had the worst stroke outcomes and an in-hospital death rate in COVID-19 positive patients vs. COVID-19 negative patients (33.3 vs. 12.9%, *p* < 0.0001); the authors hypothesized that increased stroke severity may be secondary to multiorgan involvement and more critically ill status when infected by SARS2-CoV-2 virus.

When comparing with ischemic stroke, the incidence of intracranial hemorrhage in COVID-19 related stroke is between 0.9 and 15.2% (Rothstein et al., 2020; Shahjouei et al., 2020; Yamakawa et al., 2020). This large range in incidence may be associated with the percentage of patients that were on systemic anticoagulation therapy at the onset of the stroke during their hospital stay (studies varied between prophylactic to therapeutic anticoagulation). The Dhamoon group cohort (Dhamoon et al., 2021) had 45.7% of COVID-19 patients on anticoagulation and a higher incidence of intracranial hemorrhage (68.6% in COVID-19 positive patients versus 90.7% in COVID-19 negative patients). The higher incidence in this group of patients may indicate a direct correlation between anticoagulation therapy and intercranial hemorrhage during COVID-19 infection.

On the other hand, Rothstein et al. (2020) found that in 3 Philadelphia hospitals with 844 patients admitted with COVID-19 infection, ischemic stroke was found in 2.4% of the patients and intracerebral hemorrhage was present in only 0.9% of cases. When comparing with prior observational studies, the incidence of ischemic stroke was similar to the rate reported previously in Wuhan (2.4%) (Mao et al., 2020), Italy (2.5%) (Lodigiani et al., 2020), and Netherlands (2.5%) (Klok et al., 2020). COVID-19 related stroke rates appear to be higher than rates presented in New York City; some have hypothesized that race/ethnicity may play a role in these differences, given that there is a higher proportion of black patients in the Philadelphia study (80%) and higher white population in the New York study (70%) (Yaghi et al., 2020). Whether this explanation is sufficient to explain the difference in stroke rates remains to be tested.

Dissecting the Philadelphia group data (Rothstein et al., 2020), patients developing stroke had a higher cardiovascular risk profile, as they had nearly twice the rate of hypertension and diabetes when compared with New York population. Additionally, in-hospital mortality was higher in New York than in Philadelphia (43 vs. 25%). The differences found between both populations may be related to the difference between populations, additional comorbidities, severity of illness, and compromise of the health systems during the pandemic. To summarize, COVID-19 infection begins as an upper respiratory tract disease but may quickly spread to involve multiple organs during the acute phase. Severity and extent of COVID-19 disease may vary according to attendant comorbidities and genetic, ethnic, or geographic backgrounds. Further studies delineating stroke risk in diverse populations are forthcoming.

## Stroke Mechanisms and Virchow’s Triad

Virchow’s Triad was initially used to determine the contributing factors to venous thromboembolism; however, in recent years, the observations of Virchow have been also applied to arterial thromboembolism as well. It is now widely accepted that all three limbs of Virchow’s Triad are satisfied in the arterial processes of: inflammation, endothelial dysfunction, atherosclerosis, turbulent blood flow, and abnormal function of platelet and coagulation pathways, which lead to arterial thrombosis (Jerjes-Sanchez, 2004). In this section, we review each limb of Virchow’s triad (hypercoagulable state, vascular damage, and intravascular stasis of blood) in the context of COVID-19 infection, and discuss the effects of these factors upon risk of developing stroke and other related intravascular thrombotic diseases.

### Hypercoagulable State

**FIGURE 2:**
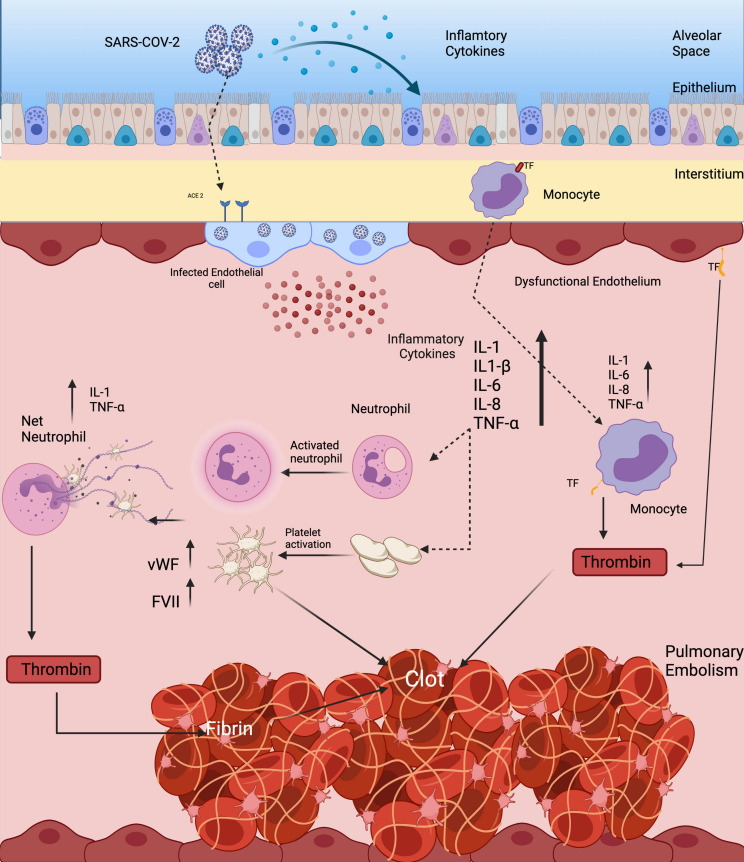
Hypercoagulable state responsible for causing stroke and PE in SARS-CoV-2 virus. Direct infection of vascular endothelial cells through the ACE2 receptor leads to expression of TF, platelet activation, and increased levels of von Willebrand Factor and Factor VIII, all of which contribute to thrombin generation and fibrin clot formation. Platelet activation facilitates the creation of neutrophil extracellular traps. All of these inflammatory changes activate monocytes and leucocytes that contribute to a pro-thrombotic cytokine storm.

Tan et al. (2020) found that there is a significant increase in overall blood clot formation in COVID-19 infected patients vs. non-infected controls. In this study, the Investigators performed a clot waveform analysis utilizing a global hemostatic assay in which higher values are related to hypercoagulability. They found that when SARS-CoV-2 virus enters pulmonary alveolar epithelium *via* the human ACE2 receptor, there is a profound change in the epithelial microenvironment associated with cytokine storm, similar to the conditions found in acute respiratory distress syndrome (ARDS). In a related study, Huang et al. (2020) found that nearly all COVID-19 infected patients have high expression levels of proinflammatory cytokines including: Interleukins (IL), IL-1β, IL-1 receptor antagonist, IL-7, IL-8, IL-9, IL-10, basic fibroblast growth factor, granulocyte colony stimulating factor, granulocyte macrophage stimulating factor, interferon gamma, interferon gamma inducible protein, monocyte chemoattractant protein, macrophage inflammatory protein-1 alpha, macrophage Inflammatory protein 1 beta, platelet derived growth factor, tumor necrosis factor alpha, and vascular endothelial growth factor (Figure 2). Concentrations of these pro-inflammatory plasma cytokines were elevated in both ICU patients and non-ICU patients versus in healthy adult controls. In addition to direct cytotoxicity, this proinflammatory state increases hypercoagulability by increasing expression of serum coagulation factors including: Tissue factor, von Willebrand factor, Factor VIII, and activation of platelets, leading to thrombin generation and fibrin clot formation (Figure 2). Thrombin, in turn, increases inflammation through its direct effect on platelets, which promote extracellular traps formation in neutrophils. It also activates endothelium through the PAR receptor, which leads to release of C5A that further activates monocytes (Abou-Ismail et al., 2020).

Additionally, in a retrospective observational study in 3 hospitals in Philadelphia Rothstein et al. (2020) reported 78% of COVID-19 patients had elevated antiphospholipid antibodies (Zhang et al., 2020), and the burden was almost exclusively anticardiolipin. In a meta-analysis by Nannoni et al. (2021), antiphospholipid antibodies were measured in 87 COVID-19 patients, and 17.2% tested positive for IgM/IgG anticardiolipin or anti-beta-glycoprotein I antibodies.

Microscopic evaluation of tissue affected by SARS-CoV-2 virus as well as from other systems affected indirectly by the virus can help clarify the mechanisms of tissue damage as well as pathologic response. Parra-Medina et al. (2021) found the presence of microthrombi in 60% of autopsies of patients with COVID-19 infection. Microthrombi were present in the lungs (73%), heart (11.2%), kidney (24%), and liver (16.3%). They found that patients with microthrombi had more comorbidities as arterial hypertension (62%), obesity or overweight (64%), diabetes mellitus type 2 (51%), and heart disease (53%). Hypertension was the strongest predictor of microthrombi in this group of patients (*p* < 0.0001) (Parra-Medina et al., 2021). These findings are suggestive of a strong correlation to activation of platelets and coagulation factors in the setting of an abnormal endothelium and vascular system as most of these risk factors are also associated with risk factors for development of atrial fibrillation and increase stroke risk as demonstrated with the CHA_2_DS_2_-VASc score that predicts stroke risk in patients without COVID-19 infection.

All these biochemical findings create the “perfect storm” for the generation of a hypercoagulable state that facilitates the formation of intravascular and intracardiac clots leading to stroke in COVID-19.

### Vascular Damage: Vasculitis and Cardiomyopathy

#### Vasculitis

COVID-19 infection can generate cutaneous vasculitis-like lesions and systemic arterial and venous thromboemboli, including cryptogenic strokes and other related forms of vasculitis. Likewise, vasculitis has been described in young or pediatric-age patients that develop cutaneous vasculitis and a systemic Kawasaki-like syndrome (McGonagle et al., 2021). In a national registry study from Spain (Galván Casas et al., 2020), 6% of the patients of a cohort 371 presented with necrotic skin lesions, the most severe presenting in older patients. In another series of cases, a 30-fold increase of Kawasaki-like diseases was found (Verdoni et al., 2020). Children present multiple symptoms of systemic inflammation and in many cases presenting cardiac involvement and features of macrophage activation syndrome (Verdoni et al., 2020). Other potential findings of systemic inflammation in children after a COVID-19 infection have been the multiple reports of patients with tachycardia, gastrointestinal symptoms, conjunctival injection mucosal changes all the symptoms accompanied by high levels of C-reactive protein, d-dimer and troponin (Dufort et al., 2020). Kawasaki-like vasculitis has been reported in a few cases (Akca et al., 2020) but the data are not yet conclusive enough to prove that the virus can directly cause inflammation of the small vessels as well; more evidence is necessary to correlate pathophysiology with the clinical findings in younger patients.

#### Neurovascular Manifestations

In a post-mortem study with microscopy and high-resolution magnetic resonance imaging of the brains of patients with COVID-19 (median age, 50 years) (Lee et al., 2021), abnormalities were seen in the brains of 10/13 patients. Magnetic resonance microscopy showed punctate hyperintensities in 9 patients, which represented areas of microvascular injury and fibrinogen leakage. These features were observed on corresponding histopathological examination performed with the use of fluorescence imaging. In 5 of these patients, thinning of the basal lamina of the endothelial cells was found. MRI findings of punctate hypo-intensities in 10 patients corresponded to congested blood vessels with surrounding areas of fibrinogen leakage and intact vasculature on autopsy. Interestingly, of these patients, SARS-CoV-2 virus was not detected by means of polymerase chain reaction with multiple primer sets, RNA sequencing of several areas of the brain, or RNA *in situ* hybridization and immunostaining. This surprising finding may be secondary to either low viral load or viral clearing from brain tissue prior to death. Alternatively, vascular damage may have directly caused these findings independently from infection of neuronal tissue. Overall, these findings demonstrate different pathways by which the SARS-CoV-2 virus can directly or indirectly affect the brain and evolve to further macroscopic damage as vessel occlusion or hemorrhage.

#### Cardiovascular Inflammation and Cardiomyopathy

Multiple studies have shown that COVID-19 infection results in significant cardiac sequelae. Early autopsy studies report elevated levels of troponin, myoglobin, C-reactive protein, serum ferritin and Interlukin-6 in ventricular myocardium (Ruan et al., 2020). These findings suggest at least two possible mechanisms for myocarditis. One theory hypothesizes that the virus produces a cytokine storm syndrome that subsequently provokes a continuous inflammatory cycle within myocardial tissue. This theory is supported by autopsy reports describing abundant interstitial mononuclear cells in the myocardium (Liu et al., 2020). Another theory suggests that pulmonary vascular compromise from an ARDS-like syndrome restricts oxygen and nutrient delivery to and from the heart, thus generating acute cardiac injury leading directly to cardiomyopathy (Nan et al., 2020). A study by Chen et al. (2020) found elevated levels of cardiac troponin and pro-brain natriuretic peptides were related with a significant increase in plasma levels of IL-6 in 120 COVID-19 infected patients. It is possible that these two mechanisms are co-existent, creating a hostile environment in the myocardium for inflammation and vascular damage. Additionally, a catecholamine surge secondary to stress in critically ill patients might contribute to myocardial damage and pump failure, causing hemodynamic instability. Currently, it is not well understood how cardiomyopathy develops; more data will be required to answer the true origin of myocardium compromise.

### Vascular Stasis

The third element of Virchow’s triad is vascular stasis. In the literature, there is not yet an abundance of data describing the extent and location of vascular stasis in COVID-19; however, several physiologic principles guide the assessment of this limb of Virchow’s triad. When endothelial damage and platelet activation occur with clot formation, there is a reduction of vessel lumen size and a reduction of vascular flow associated with stasis (Mehta et al., 2020; Munjal and Khandia, 2020). Hypercoagulable state is in part responsible for both blood clot formation and microthrombi and contributes to turbulent blood flow (Bray et al., 2020). Vascular stasis is commonly present in critically ill patients and in patients with COVID-19 infection, as both groups have an increased risk for thrombosis secondary to multiple external risk factors. Severity of the illness determines the overall level of mobility of the patient, as patients who are critically ill will have reduced functional capacity and will stay in bed more often that those who are asymptomatic. Another important associated factor is the level of care during admission, as ICU patients have an increased risk for developing deep vein thrombosis to 30% in the first week of hospital stay, and this incidence can be equal or probably higher in ICU patients admitted for COVID-19 infection (Attia et al., 2001). Also, due to the high infection rate, the COVID-19 infected patients are isolated, even when admitted in the hospital, the interaction with nurses or even physical therapy is typically minimal, and this reduces the mobility of the patient, potentially precluding rehab in the convalescent period. All these factors increase vascular stasis and increase risk of thrombosis facilitating the cascade for embolic events.

## Risk Factors for Development of Stroke

COVID-19 infected patients who develop acute cerebrovascular events are typically older (pooled median difference for age = 4.8 years; 95% CI = 1.7–22.4), and event rates were not different based on sex or gender alone (Nannoni et al., 2021). Common cardiovascular risk factors are more frequently present in COVID-19 infected patients who develop acute cerebrovascular events (Table 1). In a large meta-analysis, the common cardiovascular risk factor associated with stroke in patients with COVID-19 were found to be: hypertension [81 of 113 vs. 2392 of 11,683; OR:7.35 (95%CI: 1.94–27.87)], diabetes mellitus [52/113 vs. 1489/11,683; OR:5.56 (95%CI: 3.34–9.24)], and coronary artery disease [18/38 vs. 508/2181; OR:3.12 (95%CI: 1.61–6.02)]. There are no significant difference in rates of COVID-19 related stroke among smokers versus non-smokers [23/71 vs. 560/9374; OR:3.69 (95%CI: 0.47–29.23)]. Hypertension, diabetes mellitus and coronary artery disease are strong cardiovascular risk factors for the development of atherosclerosis, endothelial dysfunction and inflammation, and they are also associated with increased risk of stroke in patients with non-valvular atrial fibrillation as per the CHADS2-VASc score. These same risk factors were found in patients with COVID-19 infection that relate to the risk of embolic event during the inflammatory phase of the COVID-19 infection. Another important observation is the evidence of patients with stroke and COVID-19 infection without known comorbidities with an incidence of 22%; these findings suggest a higher incidence of stroke in patients without traditional risk factors (Shahjouei et al., 2021). Further studies are necessary to understand if COVID-19 infection is a trigger for stroke development in patients without know comorbidities or is just a coincidence or an indirect consequence of critical illness (Walkey et al., 2011; Moss et al., 2017).

**TABLE 1:** Cardiovascular risk factors for acute stroke in COVID-19 patients from recent studies.

	**Study**	**Patients with stroke**	**Incidence**	**Percentage**	**OR (95% CI)**	***p* value**
**Risk factor for acute stroke in patients with COVID-19**						
Hypertension	Nannoni et al., 2021	113	81/11,683	71.6	7.35 (1.94–27.87)	
	Qureshi et al., 2021	103	87/7,709	84.5		<0.0001
	Dhamoon et al., 2021	105	82/277	78.1		0.78
Diabetes mellitus	Nannoni et al., 2021	113	52/11,683	46	5.56 (3.34–9.24)	
	Qureshi et al., 2021	103	58/7,709	56.3		<0.0001
	Dhamoon et al., 2021	105	43/277	40.9		0.56
Coronary artery disease	Nannoni et al., 2021	38	18/2,181	47.36	3.12 (1.61–6.02)	
	Qureshi et al., 2021	105	20/277	19.2		0.13
Hyperlipidemia	Qureshi et al., 2021	103	78/7,709	75.7		<0.0001
	Dhamoon et al., 2021	105	47/277	44.7		0.93
Atrial Fibrillation	Qureshi et al., 2021	103	29/7,709	28.1		<0.0001
	Dhamoon et al., 2021	105	11/277	10.4		0.57
Atrial Fibrillation	Qureshi et al., 2021	103	34/7,709	33		<0.0001
	Dhamoon et al., 2021	105	13/277	12.3		0.7

*Incidence and percentage are shown among different patient cohorts with corresponding *p*-values.*

## Ischemic vs. Hemorrhagic Stroke

Ischemic stroke is the most frequent type of stroke reported in patients with COVID-19 infection. A very small group of patients were found to have hemorrhagic stroke. For patients with intracerebral hemorrhage, Nannoni et al. (2021) found that of 102 patients identified, 44.1% showed a strictly lobar hematoma and 18.5% the volume of the hematoma led to intracranial herniation. Hemorrhagic stroke has been less frequently described in association with COVID-19 infection and it might be mostly related with the use of anticoagulation in these patients as described above. Qureshi et al. (2021) found in a cohort of 85,645 patients with COVID-19 that 154 patients (0.2%) had intracerebral hemorrhage versus of 667 patients of 197,073 patients without COVID-19 (0.3%) in a data set comprising 62 health care facilities. They found a lower risk of intracerebral hemorrhage in patients with COVID-19 infection when compared with the patients without COVID-19 (odds ratio 0.5; 95% CI 0.5–0.6; *p* < 0.0001) after adjustment for sex, age, cardiovascular risk factors and long-term anticoagulation use (Qureshi et al., 2021).

In 23 studies the number of patients with SARS-CoV-2 infection was 20,850 and the number of patients with neurological manifestations was 1996 (9.5%). The total number of patients with neuroradiological abnormalities was 602 (2.8%). SARS-CoV-2 has led to various neuroimaging abnormalities (Bahranifard et al., 2021). Of these abnormalities, ischemic stroke was the most frequent with an incidence of 27%, followed by microhemorrhages 18%, imaging abnormalities without diagnosis 12%, and intraparenchymal hemorrhages in 7% of the population. In another study, a group of 58 ICU patients with COVID-19 infection were evaluated for infection-related neurological manifestations (Helms et al., 2020). The median age of the patients in this study was 63 years old. In 13 of these patients, a brain MRI was performed for unexplained encephalopathic features and they found enhancement in leptomeningeal spaces was in 8 patients, and bilateral frontotemporal hypoperfusion was noted in all 11 patients who underwent perfusion imaging. Two asymptomatic patients each had a small acute ischemic stroke with focal hyperintensity on diffusion-weighted imaging and an overlapping decreased apparent diffusion coefficient, and 1 patient had a subacute ischemic stroke with superimposed increased diffusion-weighted imaging and apparent diffusion coefficient signals. These data demonstrate the potential for occult involvement of the brain in patients with COVID-19 infection and they might present with silent strokes that can also be misdiagnosed in the setting of neuro-depressive medications, ICU stay, or with other conditions as agitation and in hospital delirium. Thus, in addition to neurovascular thrombosis, COVID-19 infection also directly affects brain parenchyma and has a direct neurotoxic effect, which may increase the pro-thrombotic milieu.

## Pulmonary Embolism

Another frequent complication in hospitalized patients with COVID-19 is pulmonary embolism (PE). Riyahi et al. (2021) in a multicenter study found an incidence of pulmonary embolism of 25% in hospitalized COVID-19 patients. Other similar studies report an incidence to be around of 1.9–8.9% of patients (Galeano-Valle et al., 2020; Grillet et al., 2020; Lodigiani et al., 2020; Stoneham et al., 2020). One possible explanation for the variation of incidence between difference studies is the condition and classification of critical ill patients and the short period of following time in the cohort studies.

The pathophysiology behind PE formation can be attributed to: hypercoagulable state, vascular damage, venous stasis and one additional factor an immunological response to decrease viral load that induces an inflammatory state followed by local activation of hemostasis that is the product of the interaction between endothelium and the platelets (Thachil and Lisman, 2020).

## Management

### Treatment

The management of stroke in patients with COVID-19 infection has been performed primarily based on current management guidelines for patients with stroke without COVID-19 infection. In ischemic stroke, the evaluation for intravenous thrombolytic therapy should be performed as in any other patient, but with taking the necessary precautions for infection control (Venketasubramanian et al., 2021). Additionally, the use of mechanical thrombectomy in selected patients should be the same as COVID-19 negative patients. Nannoni et al. (2021) showed that around 1,200 patients with acute ischemic stroke, 19.1% (236/1,205) received intravenous thrombolysis and 25.9% (238/1,223) underwent endovascular thrombectomy. Outcomes such as stroke or death in this cohort of patients were not reported; however, other studies after multivariable analysis showed no significant different in the treatment outcomes (Siegler et al., 2021). The safety of both therapies during COVID-19 infection are still being investigated, however, some anecdotal case studies have reported varying results with mechanical thrombectomy and have highlighted a possible increase of re-occlusion risk in patients with initial acute large vessel occlusion (Escalard et al., 2020; Oxley et al., 2020; Wang A. et al., 2020). Expanding knowledge of the interplay of innate immunity with atherothrombosis and stroke treatment has attracted more attention during the COVID-19 pandemic (Gerotziafas et al., 2020).

### Prevention

Guidelines on the use of anticoagulants in preventing COVID-19 related venous thromboembolism (VTE) have been heterogeneous, and there is much less information regarding anticoagulant use for prevention of stroke in this population. The American Society of Hematology suggests using prophylactic over intermediate levels of anticoagulation for patients with COVID-19 infection who are critically ill and who do not have suspected or confirmed VTE, unless there is high risk of bleeding or significant contraindications (Cuker et al., 2021). In patients previously on anticoagulation for stroke prophylaxis, it is recommended that they should continue full anticoagulation unless contraindicated. Other studies aimed to understand how beneficial prophylactic anticoagulation may be in COVID-19 patients. The ACTION trial, which evaluated the use of prophylactic versus full dose anticoagulation in hospitalized COVID-19 patients randomly assigned 615 patients who had elevated d-dimer (Lopes et al., 2021). They found that an in-hospital anticoagulation strategy with either rivaroxaban versus enoxaparin, followed by 30 days of rivaroxaban did not improve clinical outcomes, and contributed to increased bleeding, as compared with prophylactic anticoagulation alone (Lopes et al., 2021). Therefore, the use of full anticoagulation should be used only when there exists an evidence-based reason for anticoagulation, as COVID-19 by itself, even when severe, is not considered a true indication for full anticoagulation (Cuker et al., 2021; Lopes et al., 2021).

## Outcomes and Long-Term Consequences After COVID-19 Infection

A recent meta-analysis observed that out of 1,655 patients, 521 patients (31.5%) suffered in-hospital death, whereas 19.1% (376/1315) were discharged home and 25.7% (288/744) were discharged to a rehabilitation facility (Nannoni et al., 2021). Huang et al. (2021), published a retrospective six month follow-up in patients that were previously diagnosed with COVID-19 in Wuhan, China. They were able to follow 1,733 patients of the 2,469 patients discharged with COVID-19 infection (Huang et al., 2021). Of this group, only 4% was admitted in the ICU. The investigators found that 76% of the population had at least one symptom after COVID-19 infection at 6 months, and the majority of the COVID-19 survivor patients complained of fatigue or muscle weakness (63%), sleep problems (26%), anxiety or depression (23%) (Huang et al., 2021). These findings are consistent with prior investigations from previous SARS survivors which had appropriate physical recovery after a year but 33% reported a significant decrement in mental health a year later (Tansey et al., 2007). The mechanisms by which the SARS-CoV-2 virus causes mental sequelae is likely to be multifactorial and related to immunological response, direct viral infection, corticosteroid use, social isolation, stigma and ICU stay (Rogers et al., 2020). At this time, the long-lasting effects of COVID-19 in multiple organ systems affected by COVID19 are still unknown, and the National Institutes of Health has made the study of PASC a major research priority (Rogers et al., 2020).

## Cardiovascular Effects of COVID-19 Vaccines

With the rapid spread of COVID-19 and in the attempt to control further morbidity and mortality, multiple countries started with the development of a vaccine for the SARS-CoV-2 virus. During one of the most rapid vaccine trial rollouts in history, multiple vaccines were developed each with different mechanisms of action (messenger RNA, adenovirus vaccine, attenuated virus) and started clinical trials in late 2020, showing excellent results for the prevention of severe symptoms, illness and death. In 2021, there has been wide dissemination of multiple vaccines, though herd immunity remains elusive.

While the long-term effects of the vaccine are still unknown, some initial reports of possible cardiovascular effects were reported that were associated to the administration of the vaccine. Post-vaccination adverse events recorded from the United States Military and the Defense Health Agency showed that 23 male patients (22 currently serving in the military and 1 retiree) median age of 25-year-old [range 20–51] presented with acute onset of marked chest pain within 4 days after receipt of an mRNA COVID-19 vaccine. All military members were previously healthy with a high level of fitness. Seven received the BNT162b2-mRNA (Pfizer) vaccine and 16 received the mRNA-1273 (Moderna) vaccine. A total of 20 patients had symptoms following the second dose of an appropriately spaced 2-dose series. All patients had significantly elevated cardiac troponin levels. Among 8 patients who underwent cardiac magnetic resonance imaging within the acute phase of illness, all had findings consistent with the clinical diagnosis of myocarditis. Additional testing did not identify other etiologies for myocarditis, including acute COVID-19 and other infections, ischemic injury, or underlying autoimmune conditions. All patients received brief supportive care and were recovered or recovering at the time of this report.

As of July 19, 2021, Vaccine Adverse Event Reporting System (VAERS) has received 1,148 reports of myocarditis or pericarditis among people ages 30 and younger who received COVID-19 vaccine. Most cases have been reported after mRNA COVID-19 vaccination (Pfizer-BioNTech or Moderna), particularly in male adolescents and young adults. Through follow-up, including medical record reviews, CDC and FDA have confirmed 674 reports of myocarditis or pericarditis. CDC and its partners are investigating these reports to assess whether there is a relationship to COVID-19. However, despite these side effects from the vaccine, expert consensus at this time recommends that the benefits (both individual and societal) of the vaccines outweighs the risks, and thus vaccines remain the gold-standard for the prevention and spread of COVID-19.

## Conclusion

Although stroke has been recognized as a complication associated with COVID-19 infection, the correlation between traditional cardiovascular risk factors and stroke mechanisms is not well understood. COVID-19 patients are more likely to have a cryptogenic stroke, and risk of stroke in this population occurs with less underlying cardiovascular risk factors versus non-infected patients. Diagnosis of stroke in COVID-19 patients indicates a poor prognosis, and management of stroke remains the same, following usual stroke management guidelines and by modifying cardiovascular risk factors. Guidelines-based use of anti-thrombotic and anticoagulant drugs in COVID-19 patients is recommended, but the use of anticoagulation in patients with COVID-19 without evidence of embolic events is not recommended. Use of anticoagulants is guided by the known mechanisms of Virchow’s triad, whereby reduction of vascular stasis, hypercoagulability, and endothelial damage contribute to better stroke prevention and post-COVID outcomes.

## Author Contributions

All authors listed have made a substantial, direct and intellectual contribution to the work, and approved it for publication.

## Author Disclaimer

The views expressed in this article are those of the authors and do not necessarily reflect the position or policy of the Department of Veterans Affairs or the United States government.

## Conflict of Interest

The authors declare that the research was conducted in the absence of any commercial or financial relationships that could be construed as a potential conflict of interest.

## Publisher’s Note

All claims expressed in this article are solely those of the authors and do not necessarily represent those of their affiliated organizations, or those of the publisher, the editors and the reviewers. Any product that may be evaluated in this article, or claim that may be made by its manufacturer, is not guaranteed or endorsed by the publisher.

## References

[B1] Abou-Ismail M. Y. Diamond A. Kapoor S. Arafah Y. Nayak L. (2020). The hypercoagulable state in COVID-19: incidence, pathophysiology, and management. *Thromb. Res.* 194 101–115. 10.1016/j.thromres.2020.06.029 32788101 PMC7305763

[B2] Ahmed S. Zimba O. Gasparyan A. Y. (2020). Thrombosis in coronavirus disease 2019 (COVID-19) through the prism of Virchow’s triad. *Clin. Rheumatol.* 39 2529–2543. 10.1007/s10067-020-05275-1 32654082 PMC7353835

[B3] Akca U. K. Kesici S. Ozsurekci Y. Aykan H. H. Batu E. D. Atalay E. (2020). Kawasaki-like disease in children with COVID-19. *Rheumatol. Int.* 40 2105–2115. 10.1007/s00296-020-04701-6 32936318 PMC7492688

[B4] Attia J. Ray J. G. Cook D. J. Douketis J. Ginsberg J. S. Geerts W. H. (2001). Deep vein thrombosis and its prevention in critically ill adults. *Arch. Intern. Med.* 161 1268–1279. 10.1001/archinte.161.10.1268 11371254

[B5] Bahranifard B. Mehdizadeh S. Hamidi A. Khosravi A. Emami R. Mirzaei K. (2021). A review of neuroradiological abnormalities in patients with coronavirus disease 2019 (COVID-19). *Neuroradiol. J.* 19714009211029177. 10.1177/19714009211029177 [Epub ahead of print]. 34224248 PMC8819585

[B6] Bekelis K. Missios S. Ahmad J. Labropoulos N. Schirmer C. M. Calnan D. R. (2020). Ischemic stroke occurs less frequently in patients with COVID-19: a multicenter cross-sectional study. *Stroke* 51 3570–3576. 10.1161/STROKEAHA.120.031217 33106109 PMC7678670

[B7] Beyrouti R. Adams M. E. Benjamin L. Cohen H. Farmer S. F. Goh Y. Y. (2020). Characteristics of ischaemic stroke associated with COVID-19. *J. Neurol. Neurosurg. Psychiatry* 91 889–891. 10.1136/jnnp-2020-323586 32354768 PMC7231545

[B8] Bray M. A. Sartain S. E. Gollamudi J. Rumbaut R. E. (2020). Microvascular thrombosis: experimental and clinical implications. *Transl. Res.* 225 105–130. 10.1016/j.trsl.2020.05.006 32454092 PMC7245314

[B9] Chen C. Zhou Y. Wang D. W. (2020). SARS-CoV-2: a potential novel etiology of fulminant myocarditis. *Herz* 45 230–232. 10.1007/s00059-020-04909-z 32140732 PMC7080076

[B10] Cuker A. Tseng E. K. Nieuwlaat R. Angchaisuksiri P. Blair C. Dane K. (2021). American society of hematology 2021 guidelines on the use of anticoagulation for thromboprophylaxis in patients with COVID-19. *Blood Adv.* 5 872–888. 10.1182/bloodadvances.2020003763 33560401 PMC7869684

[B11] Dhamoon M. S. Thaler A. Gururangan K. Kohli A. Sisniega D. Wheelwright D. (2021). Acute cerebrovascular events with COVID-19 infection. *Stroke* 52 48–56. 10.1161/STROKEAHA.120.031668 33280551

[B12] Dufort E. M. Koumans E. H. Chow E. J. Rosenthal E. M. Muse A. Rowlands J. (2020). Multisystem inflammatory syndrome in children in New York state. *N. Engl. J. Med.* 383 347–358. 10.1056/NEJMoa2021756 32598830 PMC7346766

[B13] Escalard S. Maïer B. Redjem H. Delvoye F. Hébert S. Smajda S. (2020). Treatment of acute ischemic stroke due to large vessel occlusion with COVID-19: experience from Paris. *Stroke* 51 2540–2543. 10.1161/STROKEAHA.120.030574 32466736 PMC7282400

[B14] Galeano-Valle F. Oblitas C. M. Ferreiro-Mazón M. M. Alonso-Muñoz J. Del Toro-Cervera J. di Natale M. (2020). Antiphospholipid antibodies are not elevated in patients with severe COVID-19 pneumonia and venous thromboembolism. *Thromb. Res.* 192 113–115. 10.1016/j.thromres.2020.05.017 32425261 PMC7227496

[B15] Galván Casas C. Català A. Carretero Hernández G. Rodríguez-Jiménez P. Fernández-Nieto D. Rodríguez-Villa Lario A. (2020). Classification of the cutaneous manifestations of COVID-19: a rapid prospective nationwide consensus study in Spain with 375 cases. *Br. J. Dermatol.* 183 71–77. 10.1111/bjd.19163 32348545 PMC7267236

[B16] Gerotziafas G. T. Catalano M. Colgan M. P. Pecsvarady Z. Wautrecht J. C. Fazeli B. (2020). Guidance for the management of patients with vascular disease or cardiovascular risk factors and COVID-19: position paper from VAS-European Independent Foundation in Angiology/Vascular Medicine. *Thromb. Haemost.* 120 1597–1628. 10.1055/s-0040-1715798 32920811 PMC7869052

[B17] Grillet F. Behr J. Calame P. Aubry S. Delabrousse E. (2020). Acute pulmonary embolism associated with COVID-19 pneumonia detected with pulmonary CT angiography. *Radiology* 296 E186–E188. 10.1148/radiol.2020201544 32324103 PMC7233384

[B18] Helms J. Kremer S. Merdji H. Clere-Jehl R. Schenck M. Kummerlen C. (2020). Neurologic features in severe SARS-CoV-2 infection. *N. Engl. J. Med.* 382 2268–2270. 10.1056/NEJMc2008597 32294339 PMC7179967

[B19] Huang C. Huang L. Wang Y. Li X. Ren L. Gu X. (2021). 6-month consequences of COVID-19 in patients discharged from hospital: a cohort study. *Lancet* 397 220–232. 10.1016/S0140-6736(20)32656-8 33428867 PMC7833295

[B20] Huang C. Wang Y. Li X. Ren L. Zhao J. Hu Y. (2020). Clinical features of patients infected with 2019 novel coronavirus in Wuhan, China. *Lancet* 395 497–506. 10.1016/S0140-6736(20)30183-5 31986264 PMC7159299

[B21] Jerjes-Sanchez C. (2004). Venous and arterial thrombosis: a continuous spectrum of the same disease? *Eur. Heart J.* 26 3–4. 10.1093/eurheartj/ehi041 15615791

[B22] Klok F. A. Kruip M. van der Meer N. J. M. Arbous M. S. Gommers D. Kant K. M. (2020). Confirmation of the high cumulative incidence of thrombotic complications in critically ill ICU patients with COVID-19: an updated analysis. *Thromb. Res.* 191 148–150. 10.1016/j.thromres.2020.04.041 32381264 PMC7192101

[B23] Lee M. H. Perl D. P. Nair G. Li W. Maric D. Murray H. (2021). Microvascular injury in the brains of patients with Covid-19. *N. Engl. J. Med.* 384 481–483. 10.1056/NEJMc2033369 33378608 PMC7787217

[B24] Liu Y. Yang Y. Zhang C. Huang F. Wang F. Yuan J. (2020). Clinical and biochemical indexes from 2019-nCoV infected patients linked to viral loads and lung injury. *Sci. China Life Sci.* 63 364–374. 10.1007/s11427-020-1643-8 32048163 PMC7088566

[B25] Lodigiani C. Iapichino G. Carenzo L. Cecconi M. Ferrazzi P. Sebastian T. (2020). Venous and arterial thromboembolic complications in COVID-19 patients admitted to an academic hospital in Milan, Italy. *Thromb. Res.* 191 9–14. 10.1016/j.thromres.2020.04.024 32353746 PMC7177070

[B26] Lopes R. D. de Barros E. S. P. G. M. Furtado R. H. M. Macedo A. V. S. Bronhara B. Damiani L. P. (2021). Therapeutic versus prophylactic anticoagulation for patients admitted to hospital with COVID-19 and elevated D-dimer concentration (ACTION): an open-label, multicentre, randomised, controlled trial. *Lancet* 397 2253–2263. 10.1016/S0140-6736(21)01203-4 34097856 PMC8177770

[B27] Lopez-Leon S. Wegman-Ostrosky T. Perelman C. Sepulveda R. Rebolledo P. A. Cuapio A. (2021). More than 50 long-term effects of COVID-19: a systematic review and meta-analysis. *medRxiv* [Preprint]. 10.1101/2021.01.27.21250617 34373540 PMC8352980

[B28] Mao L. Jin H. Wang M. Hu Y. Chen S. He Q. (2020). Neurologic manifestations of hospitalized patients with coronavirus disease 2019 in Wuhan, China. *JAMA Neurol.* 77 683–690. 10.1001/jamaneurol.2020.1127 32275288 PMC7149362

[B29] Markus H. S. Brainin M. (2020). COVID-19 and stroke-a global World Stroke Organization perspective. *Int. J. Stroke* 15 361–364. 10.1177/1747493020923472 32310017 PMC11927026

[B30] McGonagle D. Bridgewood C. Ramanan A. V. Meaney J. F. M. Watad A. (2021). COVID-19 vasculitis and novel vasculitis mimics. *Lancet Rheumatol.* 3 E224–E233. 10.1016/S2665-9913(20)30420-3 33521655 PMC7832717

[B31] Mehta J. L. Calcaterra G. Bassareo P. P. (2020). COVID-19, thromboembolic risk, and Virchow’s triad: lesson from the past. *Clin. Cardiol.* 43 1362–1367. 10.1002/clc.23460 33176009 PMC7724210

[B32] Merkler A. E. Parikh N. S. Mir S. Gupta A. Kamel H. Lin E. (2020). Risk of ischemic stroke in patients with coronavirus disease 2019 (COVID-19) vs patients with influenza. *JAMA Neurol.* 77 1366–1372. 10.1001/jamaneurol.2020.2730 32614385 PMC7333175

[B33] Moss T. J. Calland J. F. Enfield K. B. Gomez-Manjarres D. C. Ruminski C. DiMarco J. P. (2017). New-onset atrial fibrillation in the critically ill. *Crit. Care Med.* 45 790–797. 10.1097/CCM.0000000000002325 28296811 PMC5389601

[B34] Munjal A. Khandia R. (2020). Atherosclerosis: orchestrating cells and biomolecules involved in its activation and inhibition. *Adv. Protein Chem. Struct. Biol.* 120 85–122. 10.1016/bs.apcsb.2019.11.002 32085889

[B35] Nan J. Jin Y. B. Myo Y. Zhang G. (2020). Hypoxia in acute cardiac injury of coronavirus disease 2019: lesson learned from pathological studies. *J. Geriatr. Cardiol.* 17 221–223. 32362921 10.11909/j.issn.1671-5411.2020.04.010 PMC7189261

[B36] Nannoni S. de Groot R. Bell S. Markus H. S. (2021). Stroke in COVID-19: a systematic review and meta-analysis. *Int. J. Stroke* 16 137–149. 10.1177/1747493020972922 33103610 PMC7859578

[B37] Oxley T. J. Mocco J. Majidi S. Kellner C. P. Shoirah H. Singh I. P. (2020). Large-vessel stroke as a presenting feature of Covid-19 in the young. *N. Engl. J. Med.* 382:e60. 10.1056/NEJMc2009787 32343504 PMC7207073

[B38] Parra-Medina R. Herrera S. Mejia J. (2021). Systematic review of microthrombi in COVID-19 autopsies. *Acta Haematol.* 144 476–483. 10.1159/000515104 33873184 PMC8089413

[B39] Qureshi A. I. Baskett W. I. Huang W. Myers D. Lobanova I. Ishfaq M. F. (2021). Intracerebral hemorrhage and coronavirus disease 2019 in a cohort of 282,718 hospitalized patients. *Neurocrit. Care* 1–7. 10.1007/s12028-021-01297-y [Epub ahead of print]. 34231186 PMC8260011

[B40] Riyahi S. Dev H. Behzadi A. Kim J. Attari H. Raza S. I. (2021). Pulmonary embolism in hospitalized patients with COVID-19: a multicenter study. *Radiology* 210777. 10.1148/radiol.2021210777 [Epub ahead of print]. 34254850 PMC8294351

[B41] Rogers J. P. Chesney E. Oliver D. Pollak T. A. McGuire P. Fusar-Poli P. (2020). Psychiatric and neuropsychiatric presentations associated with severe coronavirus infections: a systematic review and meta-analysis with comparison to the COVID-19 pandemic. *Lancet Psychiatry* 7 611–627. 10.1016/S2215-0366(20)30203-0 32437679 PMC7234781

[B42] Rothstein A. Oldridge O. Schwennesen H. Do D. Cucchiara B. L. (2020). Acute cerebrovascular events in hospitalized COVID-19 patients. *Stroke* 51 e219–e222. 10.1161/STROKEAHA.120.030995 32684145 PMC7386677

[B43] Ruan Q. Yang K. Wang W. Jiang L. Song J. (2020). Clinical predictors of mortality due to COVID-19 based on an analysis of data of 150 patients from Wuhan, China. *Intensive Care Med.* 46 846–848. 10.1007/s00134-020-05991-x 32125452 PMC7080116

[B44] Shahjouei S. Anyaehie M. Koza E. Tsivgoulis G. Naderi S. Mowla A. (2021). SARS-CoV-2 is a culprit for some, but not all acute ischemic strokes: a report from the multinational COVID-19 stroke study group. *J. Clin. Med.* 10:931. 10.3390/jcm10050931 33804307 PMC7957755

[B45] Shahjouei S. Naderi S. Li J. Khan A. Chaudhary D. Farahmand G. (2020). Risk of stroke in hospitalized SARS-CoV-2 infected patients: a multinational study. *EBioMedicine* 59:102939. 10.1016/j.ebiom.2020.102939 32818804 PMC7429203

[B46] Siegler J. E. Zha A. M. Czap A. L. Ortega-Gutierrez S. Farooqui M. Liebeskind D. S. (2021). Influence of the COVID-19 pandemic on treatment times for acute ischemic stroke. *Stroke* 52 40–47. 10.1161/STROKEAHA.120.032789 33250041 PMC7934334

[B47] Stoneham S. M. Milne K. M. Nuttall E. Frew G. H. Sturrock B. R. Sivaloganathan H. (2020). Thrombotic risk in COVID-19: a case series and case-control study. *Clin. Med.* 20 e76–e81. 10.7861/clinmed.2020-0228 32423903 PMC7385762

[B48] Tan C. W. Low J. G. H. Wong W. H. Chua Y. Y. Goh S. L. Ng H. J. (2020). Critically ill COVID-19 infected patients exhibit increased clot waveform analysis parameters consistent with hypercoagulability. *Am. J. Hematol.* 95 E156–E158. 10.1002/ajh.25822 32267008 PMC7262023

[B49] Tansey C. M. Louie M. Loeb M. Gold W. L. Muller M. P. de Jager J. (2007). One-year outcomes and health care utilization in survivors of severe acute respiratory syndrome. *Arch. Intern. Med.* 167 1312–1320. 10.1001/archinte.167.12.1312 17592106

[B50] Thachil J. Lisman T. (2020). Pulmonary megakaryocytes in coronavirus disease 2019 (COVID-19): roles in thrombi and fibrosis. *Semin. Thromb. Hemost.* 46 831–834. 10.1055/s-0040-1714274 32882717 PMC7653545

[B51] Venketasubramanian N. Anderson C. Ay H. Aybek S. Brinjikji W. de Freitas G. R. (2021). Stroke care during the COVID-19 pandemic: international expert panel review. *Cerebrovasc. Dis.* 50 245–261. 10.1159/000514155 33756459 PMC8089455

[B52] Verdoni L. Mazza A. Gervasoni A. Martelli L. Ruggeri M. Ciuffreda M. (2020). An outbreak of severe Kawasaki-like disease at the Italian epicentre of the SARS-CoV-2 epidemic: an observational cohort study. *Lancet* 395 1771–1778. 10.1016/S0140-6736(20)31103-X 32410760 PMC7220177

[B53] Walkey A. J. Wiener R. S. Ghobrial J. M. Curtis L. H. Benjamin E. J. (2011). Incident stroke and mortality associated with new-onset atrial fibrillation in patients hospitalized with severe sepsis. *JAMA* 306 2248–2254. 10.1001/jama.2011.1615 22081378 PMC3408087

[B54] Wang A. Mandigo G. K. Yim P. D. Meyers P. M. Lavine S. D. (2020). Stroke and mechanical thrombectomy in patients with COVID-19: technical observations and patient characteristics. *J. Neurointerv. Surg.* 12 648–653. 10.1136/neurintsurg-2020-016220 32451359

[B55] Wang Z. Yang Y. Liang X. Gao B. Liu M. Li W. (2020). COVID-19 associated ischemic stroke and hemorrhagic stroke: incidence, potential pathological mechanism, and management. *Front. Neurol.* 11:571996. 10.3389/fneur.2020.571996 33193019 PMC7652923

[B56] World Health Organization (2021). *WHO Coronavirus (COVID-19) Dashboard. COVID 19 Situation by Region, Country, Territory & Area Updated by WHO.* Available online at: https://covid19.who.int/2021 (accessed October 25, 2021).

[B57] Yaghi S. Ishida K. Torres J. Mac Grory B. Raz E. Humbert K. (2020). SARS-CoV-2 and stroke in a New York healthcare system. *Stroke* 51 2002–2011. 10.1161/STROKEAHA.120.030335 32432996 PMC7258764

[B58] Yamakawa M. Kuno T. Mikami T. Takagi H. Gronseth G. (2020). Clinical characteristics of stroke with COVID-19: a systematic review and meta-analysis. *J. Stroke Cerebrovasc. Dis.* 29:105288. 10.1016/j.jstrokecerebrovasdis.2020.105288 32992199 PMC7456266

[B59] Zhang Y. Xiao M. Zhang S. Xia P. Cao W. Jiang W. (2020). Coagulopathy and antiphospholipid antibodies in patients with Covid-19. *N. Engl. J. Med.* 382:e38. 32268022 10.1056/NEJMc2007575 PMC7161262

